# Mycologically confirmed chronic pulmonary aspergillosis in a post-pulmonary tuberculosis patient in Ghana

**DOI:** 10.4314/gmj.v56i4.13

**Published:** 2022-12

**Authors:** Bright K Ocansey, Abraham Adjei, David W Denning

**Affiliations:** 1 Division of Evolution, Infection and Genomics, Faculty of Biology, Medicine and Health, University of Manchester, Manchester, UK; 2 Chest Clinic, Department of Medicine and Therapeutics, Korle-Bu Teaching Hospital, Accra, Ghana

**Keywords:** tuberculosis, pulmonary, aspergillosis, Ghana, mycologically-confirmed

## Abstract

Pulmonary tuberculosis (PTB) remains a major public health challenge in low- and middle-income countries. PTB may leave residual cavitation following treatment in some patients, allowing saprophytic colonization by Aspergillus species, resulting in a slow, progressive lung condition known as chronic pulmonary aspergillosis (CPA). PTB is the commonest underlying condition in CPA, mainly post-treatment. CPA is likely to be misdiagnosed as PTB reactivation due to clinical and radiological similarities. Ghana has a significant PTB burden, but only one case of clinically and radiologically diagnosed CPA has been reported, and epidemiological studies are also lacking. The definitive diagnosis of CPA comprises symptomatology, imaging findings and mycological evidence. Mycological evidence is critical to rule out other differential diagnoses, including non-*Aspergillus* pulmonary fungal infections and has implications for treatment choice. Herein, we present a case of mycologically-confirmed CPA in a previously treated PTB patient.

**Funding:**

Fungal laboratory testing was provided by a CARIGEST SA studentship and research award to BKO and DWD respectively.

## Introduction

Pulmonary tuberculosis (PTB) remains a major public health challenge, especially in low- and middle-income countries. Patients previously treated for PTB are susceptible to post-TB complications making long-term outcomes very important.^1^ PTB may leave residual cavitation following treatment and allow saprophytic colonization of the ubiquitous fungus *Aspergillus* when its spores are inhaled. This causes the expansion of existing cavities or creates new ones and may be accompanied by symptoms resulting in a slow, progressive lung condition known as chronic pulmonary aspergillosis (CPA). Almost all CPA cases have an underlying lung condition, but PTB is the commonest.^2^ CPA incidence is reported to be between 4.6% - 22% in post-TB patients, with a global prevalence of 1.74 million.^2^–^6^ There are several differential diagnoses of CPA, including PTB, non-tuberculous mycobacterial infection, pulmonary hydatid disease, lung cancer and other pulmonary fungal infections.^7^,^8^ Thus, establishing mycological evidence by demonstrating immunological response to *Aspergillus* and/or detecting the presence of *Aspergillus* by culture or direct microscopy is critical for a definitive diagnosis of CPA.^7^,^8^

In the last decades, only one case of CPA has been reported in Ghana, where the diagnosis was based on imaging findings.^9^ In the current report, we present a case of mycologically-confirmed CPA in a previously treated PTB patient in Ghana. We aim to raise awareness about CPA and to showcase the relevance of an *Aspergillus*-specific IgG and IgM lateral flow assay (LFA) in CPA diagnosis.

## Case Report

### Clinical history

In January 2021, a 27-year-old male student was reviewed at the Chest Clinic of the Korle-Bu Teaching Hospital, Accra, Ghana, as a recurrent PTB with severe cough and breathlessness. His medical history showed he was HIV-negative and was diagnosed and treated for PTB in 2008. He had also been hospitalised for recurrent productive cough and haemoptysis episodes in 2017 and 2020. The patient indicated he had not resorted to any other treatment, including herbal medicine.

### Investigations

**Figure 1:**
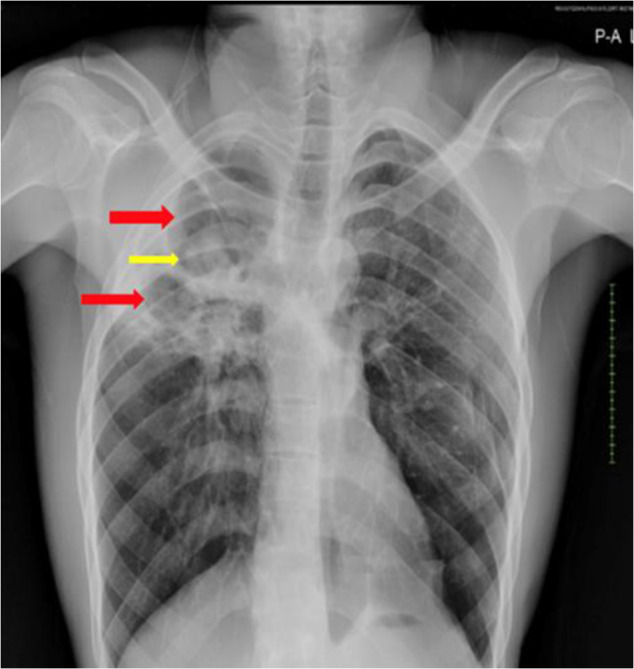
Patient's chest radiograph showing thin-walled right upper lung zone cavities (red arrows) with a soft tissue density lesion (probably aspergilloma) within the dependent portion of the superior cavity (yellow arrow). An air crescent sign (Monod's sign) is seen surrounding another aspergilloma in the smaller cavity close to the right hilum.

The molecular test for *Mycobacterium tuberculosis* (Xpert MTB/RIF) was negative. Chest radiograph showed three thin-walled cavities in the right upper lobe with a dependent intracavitary soft tissue mass or fungal ball, probably aspergilloma in two cavities and surrounding fibrosis (Figure 1). Computed tomography (CT) scan reported multiple thin-walled right upper lobe cavitary lesions with an intracavitary soft tissue mass and bilateral upper lobe fibrosis with associated traction bronchiectasis, indicative of a background of post-primary PTB. Bacterial culture of sputum grew *Pseudomonas aeruginosa. Aspergillus*-specific IgG and IgM LFA (LDBio Diagnostics, Lyon, France) were positive and fungal culture of sputum grew *Aspergillus fumigatus* and *Aspergillus niger*. On inquiring, the patient was not living in a damp house, involved in traditional (firewood or charcoal) cooking, farming or gardening activity, and had no history of smoking which are all potential epidemiological risk factors for aspergillosis.^5^ A diagnosis of CPA with bacterial co-infection was made.

### Treatment

The patient was started on oral voriconazole, 200mg twice daily and placed on levofloxacin, 500mg twice daily for bacterial co-infection for two weeks.

### Follow up

After two weeks, the patient was symptom-free. Due to the high cost of voriconazole, treatment was switched to oral itraconazole, 200mg twice daily for two weeks and 200mg daily. The patient remained asymptomatic on itraconazole for six months and was lost to follow-up. However, seven months later, he was reached via phone and was doing well with no symptoms.

### Ethical Statement

Informed consent was obtained from the patient for the anonymous publication of this report, and confidentiality was assured.

## Discussion

Epidemiological data of CPA in post-TB patients is gradually improving in Africa, especially in countries with high TB burden countries.^10^ In Ghana, a deterministic modelling study estimated an annual CPA incidence of 2002.^11^ Definitive diagnosis of CPA is generally tricky and involves a combination of clinical symptoms, characteristic imaging findings and laboratory tests, including serology and microbiology to demonstrate immunological responses to and/or presence of *Aspergillus* spp. 7,8 Development of cavities occurs in about 20-40% of PTB patients susceptible to *Aspergillus* local infection.^2^ Although CPA usually complicates PTB treatment, it may occur concurrently with an active PTB infection and be initiated towards the end of PTB therapy.^8^,^12^ The similarities in clinical manifestations and imaging findings make CPA likely to be misdiagnosed as PTB in most TB endemic regions where mycology laboratories are unavailable.^13^–^15^

*Aspergillus*-specific IgG and/or IgM is the most reliable diagnostic test for CPA, and elevated levels are reported in 95-100% of all cases.^8^ However, a positive *Aspergillus* antibody test is not specific and may also be observed in conditions such as allergic bronchopulmonary aspergillosis (ABPA), *Aspergillus* bronchitis, subacute invasive aspergillosis, and community-acquired *Aspergillus* pneumonia.^16^ In our case, we used the LDBio *Aspergillus* IgG and IgM LFA with comparable high performance to the recommended *Aspergillus*-specific IgG immunoassays, which have recently been used clinically in Uganda.^6^,^17^,^18^ Careful interpretation of *Aspergillus* IG is required in immunocompromised hosts as their ability to mount detectable antibody response decreases.^5^,^19^ The sensitivity of traditional sputum culture is low but may be improved by using a high volume of sample, which we employed in our case.^20^

The management of CPA depends on radiological phenotypes, that is, chronic cavitary pulmonary aspergillosis and chronic fibrosing pulmonary aspergillosis require long-term antifungal therapy (at least six months).^7^ A recent randomized controlled trial in India demonstrated that 12 months of oral itraconazole was superior to a 6-month regimen in reducing relapses of CPA at two years.^21^ Itraconazole and voriconazole are the current standards for antifungal treatment, as both have acceptable tolerability and minimal toxicity.^22^

## Conclusion

This current case report highlights a probably low index of suspicion for pulmonary fungal infections such as CPA in Ghana. Early diagnosis of CPA, a major post-TB complication, can be achieved using the *Aspergillus* IgG and IgM LFA, especially among patients with recurrent TB-like symptoms without any significant improvement to standard PTB treatment.
